# Immune Pathway and Gene Database (IMPAGT) Revealed the Immune Dysregulation Dynamics and Overactivation of the PI3K/Akt Pathway in Tumor Buddings of Cervical Cancer

**DOI:** 10.3390/cimb44110350

**Published:** 2022-10-23

**Authors:** Yeseul Choi, Nora Jee-Young Park, Tan Minh Le, Eunmi Lee, Donghyeon Lee, Hong Duc Thi Nguyen, Junghwan Cho, Ji-Young Park, Hyung Soo Han, Gun Oh Chong

**Affiliations:** 1 Department of Biomedical Science, Graduate School, Kyungpook National University, Daegu 41944, Korea; 2 BK21 Four Program, School of Medicine, Kyungpook National University, Daegu 41944, Korea; 3 Department of Pathology, School of Medicine, Kyungpook National University, Daegu 41944, Korea; 4 Department of Pathology, Kyungpook National University Chilgok Hospital, Daegu 41404, Korea; 5 Clinical Omics Institute, Kyungpook National University, Daegu 41405, Korea; 6 Department of Physiology, School of Medicine, Kyungpook National University, Daegu 41944, Korea; 7 Department of Obstetrics and Gynecology, School of Medicine, Kyungpook National University, Daegu 41944, Korea; 8 Department of Obstetrics and Gynecology, Kyungpook National University Chilgok Hospital, Daegu 41404, Korea

**Keywords:** tumor budding, cervical cancer, RNA sequencing, differentially expressed genes, Gene Ontology, tumor immune microenvironment, immune profiling, immune database, PI3K/Akt signaling pathway

## Abstract

Tumor budding (TB) is a small cluster of malignant cells at the invasive front of a tumor. Despite being an adverse prognosis marker, little research has been conducted on the tumor immune microenvironment of tumor buddings, especially in cervical cancer. Therefore, RNA sequencing was performed using 21 formalin-fixed, paraffin-embedded slides of cervical tissues, and differentially expressed genes (DEGs) were analyzed. Immune Pathway and Gene Database (IMPAGT) was generated for immune profiling. “Pathway in Cancer” was identified as the most enriched pathway for both up- and downregulated DEGs. Kyoto Encyclopedia of Genes and Genomes Mapper and Gene Ontology further revealed the activation of the PI3K/Akt signaling pathway. An IMPAGT analysis revealed immune dysregulation even at the tumor budding stage, especially in the PI3K/Akt/mTOR axis, with a high efficiency and integrity. These findings emphasized the clinical significance of tumor buddings and the necessity of blocking the overactivation of the PI3K/Akt/mTOR pathway to improve targeted therapy in cervical cancer.

## 1. Introduction

Tumor budding (TB) is the formation of a cluster of fewer than five malignant cells at the invasive edge of a tumor [1,2,3]. First established in colorectal cancer, tumor budding is believed to be related to the epithelial–mesenchymal transition (EMT), which involves the loss of cell–cell connectivity along with epithelial markers, such as E-cadherin, and the gain of mesenchymal markers, such as N-cadherin or vimentin [4,5,6,7]. After morphologically transitioning, tumor cells gain a locomotive ability, allowing them to migrate and invade secondary sites and eventually leading to metastasis. The newly developed disseminated tumor cells undergo another transition called the mesenchymal–epithelial transition, which recapitulates the pathology of primary epithelial tumors and promotes tumor formation at the attached site. Briefly, TB, which exhibits EMT traits, is associated with tumor initiation, invasion, metastasis, and overall cancer progression, and it is a clinically adverse prognosis marker [3,8,9,10,11,12,13].

Despite its clinical significance, the tumor immune microenvironment (TIME) of TB has not been extensively studied, especially in cervical cancer. To date, immune gene databases such as ImSig (Immune Cell Gene Signatures) [14] or platforms such as CIBERSORT [15] or TIMER [16] have been used for immune profiling. However, these platforms do not provide a list of genes involved in specific immune pathways. Moreover, CIBERSORT only presents relative cell expression, which makes it difficult to comprehend the degree of differential gene expression.

Therefore, we used a slightly different approach than traditional analysis methods. Instead of using diverse databases to infer biological implications and deduce potential clinically meaningful biomarkers in cancer etiology, we generated a new immune database, namely, Immune Pathway and Gene Database (IMPAGT), based on the gene set of CIBERSORT. Using IMPAGT, the tumor immune microenvironment of tumor buddings in cervical cancer was investigated.

## 2. Materials and Methods

### 2.1. Patient Selection

Following approval by the Institutional Review Board of Kyungpook National University Chilgok Hospital (approval no.: 2020-10-003), 21 patients with cervical cancer were selected for the study. Cervical cancer tissue from each patient was collected after radical hysterectomy with pelvic and/or para-aortic lymphadenectomy to treat early-stage and locally advanced cervical cancer. All patients were clinically staged according to the revised 2009 International Federation of Gynecologic Obstetrics staging system for cervical cancer [17].

### 2.2. Pathological Process and Tumor Budding Counts

All cervical tissue specimens were collected after biopsy, and formalin-fixed, paraffin-embedded (FFPE) slides were generated. The FFPE slides were stained with hematoxylin and eosin (H&E), and multiple sections of H&E-stained slides were examined by pathologists at Kyungpook National University Chilgok Hospital. Based on the criterion used in a previous study (high tumor budding, ≥3 tumor buddings), among 21 cervical cancer samples, 15 had high tumor budding, whereas the remaining samples had low tumor budding [18]. The medical records of all patients were reviewed, and their clinicopathologic parameters were collected. The patient information is described in Table 1.

### 2.3. RNA Sequencing

RNA was extracted from FFPE slides of 21 patients using the ReliaPrep™ FFPE Total RNA Miniprep kit (Promega, Madison, WI, USA). Extracted RNA was then synthesized into cDNA using the TruSeq RNA Exome Kit (Illumina, San Diego, CA, USA), and an RNA sequencing dataset was generated using the Nova 6000 platform (Illumina). The raw data were quantified using the STAR [19] and RSEM [20] quantification tools, and differentially expressed gene (DEG) analysis was performed using DESeq2 [21]. The threshold for identifying DEGs was adjusted to absolute log2 fold change ≥ 1 and *p* < 0.05. The DEGs were plotted in a volcano plot graph using the “*ggplot*” package in R.

### 2.4. CIBERSORT and LM22

CIBERSORT is a computational platform designed by the Alizadeh Laboratory at Stanford University (Palo Alto, CA, USA) that uses the machine learning technique, support vector regression, to deconvolute and characterize diverse cell types from bulk sequencing data [15]. The platform requires two input data files for analysis: (1) a vector consisting of gene expression profiles (GEPs) and (2) a “signature matrix” containing signature genes for the cell subset of interest.

For immune-related analysis, LM22 was used as a signature matrix. According to Alizadeh et al., LM22 contains 547 genes that distinguish 22 mature human hematopoietic populations isolated from peripheral blood or in vitro culture conditions, including seven T cell types, naïve and memory B cells, plasma cells, NK cells, and myeloid subsets. CIBERSORT analysis using this LM22 matrix provides proportional gene expression measures of the input GEP file corresponding to 22 distinct immune cell types [15].

The 547 gene symbols of LM22 are listed in Table S1.

### 2.5. Generation of IMPAGT

STRINGdb [22] is a database that provides protein–protein interaction (PPI) networks with functional enrichment analysis using diverse functional classification tools, such as Gene Ontology (GO) and Kyoto Encyclopedia of Genes and Genomes (KEGG) [23]. For our analysis, all 547 gene symbols of LM22 were input into the “Multiple proteins” tab of STRINGdb (version 11.5) using “Homo sapiens” as the corresponding organism. All outputted KEGG pathways were retrieved. Each pathway was individually searched using the KEGG pathway database webpage (https://www.genome.jp/kegg/pathway.html (accessed on 13 June 2022)), and all corresponding genes were collected using the R package “KEGGREST”. An LM22-based IMPAGT was generated, with the KEGG pathways of LM22 presented in columns and the corresponding gene symbols presented in rows.

### 2.6. Intersection of DEGs in Tumor Budding with IMPAGT

DEGs overlapping in tumor budding and IMPAGT were identified using the basic R function “intersect”. Then, the intersecting DEGs were further searched using IMPAGT, and upregulated (orange) and downregulated (blue) DEGs were highlighted. Subsequently, the number of highlighted DEGs in each pathway was counted.

### 2.7. Enriched Pathway Visualization

Pathways with more than 10 DEGs were considered significantly enriched in the TB environment of cervical cancer. Among these pathways, the most enriched pathway was visualized using several pathway visualization tools, such as KEGG Mapper Color [24], DAVID [25], and ShinyGo [26]. The final visualization result was obtained from KEGG Mapper.

From the KEGG Mapper Color website (https://www.genome.jp/kegg/mapper/color.html (accessed on 20 June 2022)), “hsa” was selected as the search mode to identify human-specific pathways. The lists of DEGs were uploaded, with upregulated DEGs colored red and downregulated DEGs colored cyan.

### 2.8. Functional Enrichment Analysis Using DAVID

GO analysis was performed using the DAVID database (https://david.ncifcrf.gov/tools.jsp (accessed on 20 July 2022)) [25,27]. In total, 133 upregulated DEGs that overlapped with IMPAGT genes were entered as a gene list of interest, and “OFFICIAL_GENE_SYMBOL” was selected as the identifier. Using “Homo Sapiens; 9606” as the species, the gene list was analyzed. In the GO analysis, only biological process (BP) terms were retrieved.

## 3. Results

### 3.1. DEGs in Samples with High or Low Tumor Budding

**Figure 1:**
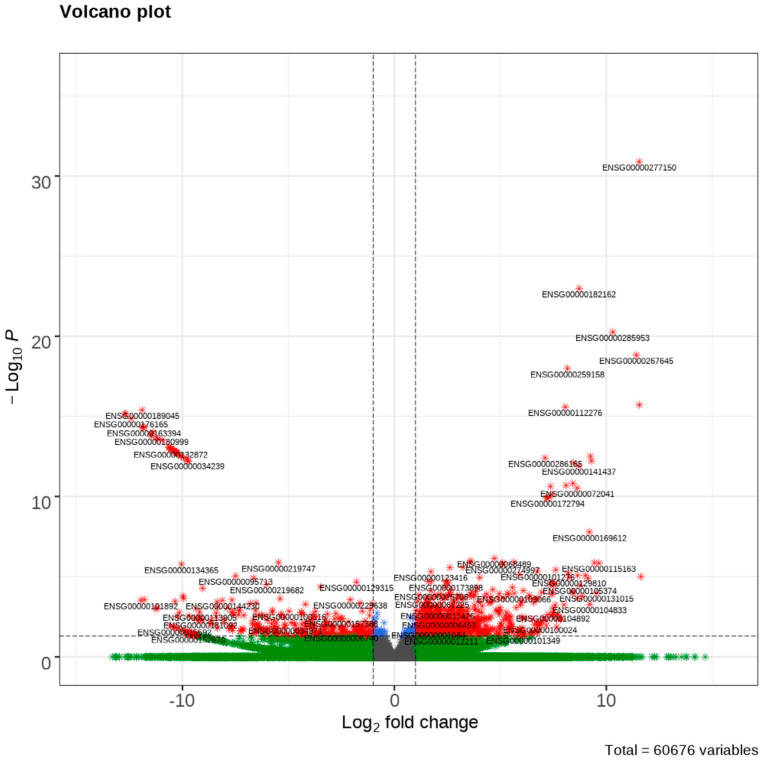
A volcano plot of differentially expressed genes (DEGs) of high tumor budding in cervical cancer. Top 30 up-regulated and top 30 downregulated DEGs are plotted. The upper right corner indicates over-expressed DEGs and upper left corner indicates down-expressed DEGs.

The RNA sequencing revealed 1253 DEGs between patients with high tumor budding and those with low tumor budding. Among these DEGs, 903 genes were upregulated and 349 were downregulated. A volcano plot of the top 30 up- and downregulated DEGs is presented in Figure 1.

### 3.2. Creation of IMPAGT

**Figure 2:**
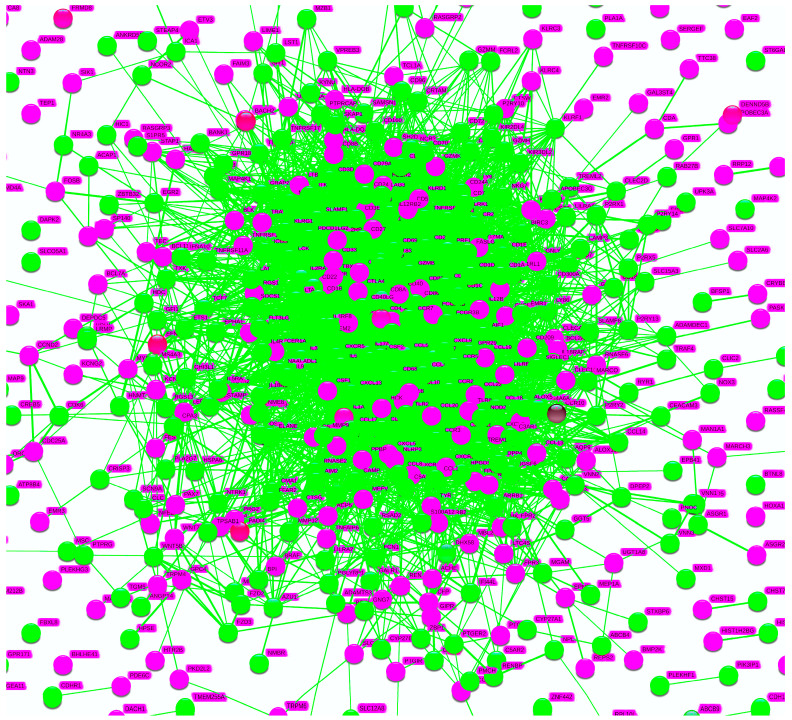
Protein–protein interaction (PPI) network of LM22 on STRINGdb. STRINGdb analysis of LM22 presented a cluster that is very interrelated, an almost ball-shaped aggregate. In total, 51 pathways were identified which were all related to immune reactions of the human body.

When the 547 genes of LM22 were analyzed in STRINGdb, the resultant PPI network was closely interrelated, forming an intricate ball cluster (Figure 2). There were 519 nodes and 6049 edges with significant PPI enrichment values of less than 1.0 × 10^−16^.

STRINGdb also identified the KEGG pathways associated with genes in the LM22 dataset. In total, 51 pathways from the KEGG database were analyzed, all of which were related to immune reactions of the human body. From these pathways, those related to direct viral infection, such as influenza A pathways, or those irrelevant to tumor budding, such as asthma pathways, were excluded, leaving 26 immune pathways. Meanwhile, 4131 genes were collected from the 26 pathways using *KEGGREST*. When overlapping genes were filtered, 2534 genes were left. This list of 2534 genes was considered the “IMPAGT gene set” which could be used for immune analysis. IMPAGT and the IMPAGT gene set are presented in Table S2.

### 3.3. Intersection of DEGs with the IMPAGT gene set

In total, 133 upregulated DEGs overlapped with the IMPAGT gene set, and they were engaged in all 26 immune pathways. Considering pathways with more than 10 DEGs as significantly enriched pathways, seven significantly enriched pathways were associated with upregulated DEGs: “Pathway in Cancer (Hsa 05200)”, “Antigen processing and presentation (Hsa 04612)”, “Transcriptional misregulation in Cancer (Hsa 05202)”, “MAPK signaling pathway (Hsa 04010)”, “NOD-like receptor signaling pathway (Hsa 04621)”, “Cell adhesion molecules (Hsa 04514)”, and “Cytokine-cytokine receptor interaction (Hsa 04060)”.

Among 31 downregulated DEGs, there were 31 overlapping DEGs, which were involved in 17 different immune pathways. The only significantly enriched pathway was “Pathway in Cancer”, and the remaining pathways contained fewer than five DEGs.

To summarize, “Pathway in Cancer” was the most strongly enriched pathway, indicating that it was the most strongly activated and dysregulated pathway in our sample. It was considered to be the most significantly enriched pathway in the TB environment of cervical cancer.

The significantly enriched pathways for both upregulated and downregulated DEGs, along with the number of DEGs involved in each pathway, are listed in Table 2.

### 3.4. Pathway Visualization Revealed Specific Enrichment of the PI3K/Akt Signaling Pathway

“Pathway in Cancer” was selected as the most significantly enriched pathway. Subsequently, it was visualized using KEGG Mapper Color. To clarify the overall involvement of TB DEGs in the pathway, all 1253 DEGs were inserted into KEGG Mapper.

**Figure 3:**
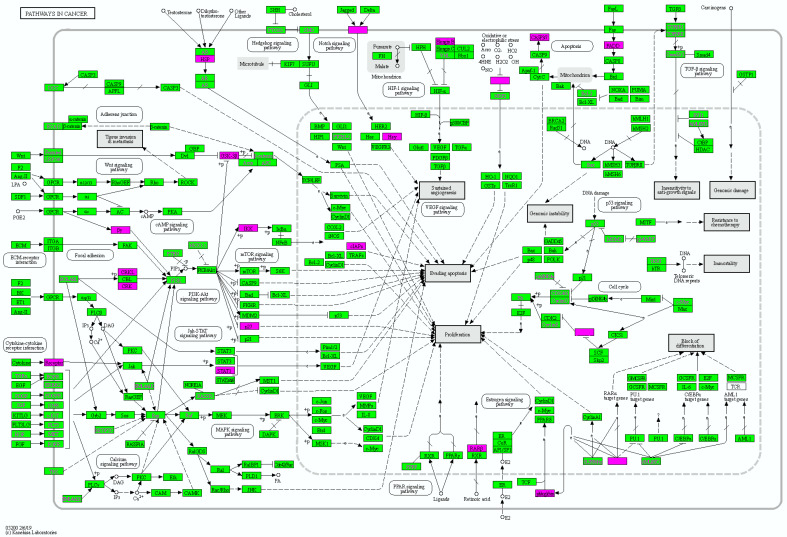
Enriched pathway visualization with KEGG Mapper Color. DEG-mapped pathway visualization presented that DEGs were mainly involved in “Signal transduction metabolism”, such as the PI3K/Akt, JAK/STAT, and Notch signaling pathways, and were specifically enriched in “PI3K/Akt signaling pathway”. All pathways were directed towards “Sustained Angiogenesis”, “Evading Apoptosis”, and “Proliferation”.

The DEG-mapped “Pathway in Cancer” revealed the points at which the upregulated and downregulated DEGs engaged the pathway (Figure 3). Most DEGs were mainly involved in the “Signaling molecules and interaction” and “Signal transduction” metabolism pathways, such as the PI3K/Akt, JAK/STAT, MAPK, and Notch signaling pathways. Because most DEGs were involved in the PI3K/Akt pathway, this pathway was examined in more detail. All signal transduction pathways were directed to “Sustained Angiogenesis”, “Evading apoptosis”, and “Proliferation”.

### 3.5. GO Analysis of 133 Overlapping Upregulated DEGs

GO analysis on 133 upregulated DEGs that intersected with IMPAGT identified BP functions closely related to the PI3K/AKT pathway. When all significant BP terms were hierarchically ordered by percentage relative to the total input genes with *p*-values of <0.05, ”Signal transduction (GO: 0007165)” and “Immune response (GO: 0006955)” were the top two BP terms. Other significantly enriched BP terms included “Positive regulation of cell proliferation (GO: 0008284)”, “Positive regulation of apoptotic process (GO: 0043065)”, “Regulation of apoptotic process (GO: 0042981)”, “Regulation of cell proliferation (GO: 0042127)”, and “Positive regulation of protein phosphorylation (GO: 0001934)” (Table 3).

**Figure 4:**
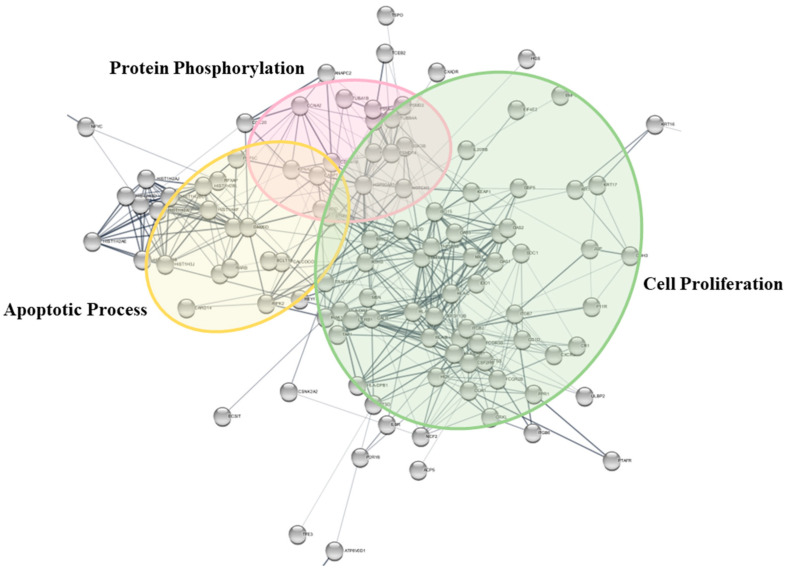
BP clusters of 133 upregulated DEGs overlapped with IMPAGT. When PPI network was clustered with BP traits, it presented a tendency to cluster into three groups: “Apoptotic process”, “Protein phosphorylation”, and “Cell proliferation”.

Meanwhile, the 133 overlapping DEGs were further visualized using STRINGdb, and the PPI network revealed a tendency to cluster into three groups, which were termed “Apoptotic process”, “Protein Phosphorylation”, and ”Cell proliferation” (Figure 4).

## 4. Discussion

This study aimed to investigate the tumor immune microenvironment (TIME) of TB in cervical cancer using the newly generated immune database IMPAGT. For immune profiling analyses, researchers mainly use immune gene databases, such as ImSig [14] or ImmuCellDB [28]. The other popular approach is to use bioinformatics applications, such as CIBERSORT [15], a deconvolution tool that characterizes immune cell compositions. However, all of these methods have limitations. First, the usage of several different databases for data interpretation is extremely time-consuming, and this is associated with a high risk of bias. Second, although CIBERSORT allows users to estimate the immune cell composition, it only provides proportional values of cell composition that are summed in one, thereby making it difficult to identify the differential expression of the input genes. Moreover, it does not provide lists of genes involved in specific immune pathways.

Considering these limitations, our study holds significance in terms of several aspects. First, IMPAGT allows researchers to interpret their data with high efficiency and integrity and a low risk of bias. IMPAGT, the “two in one” immune database, provides immune pathways and the corresponding genes of each pathway simultaneously. The integrated database enables users to investigate their genes of interest in a more rapid and efficient manner than individually searching and merging separate results from multiple databases. Furthermore, instead of obtaining “one gene–one pathway” associations based on the researcher’s interpretation, IMPAGT provides several immune pathways in which certain genes of interest are involved. In particular, it proposes “one gene–many immune pathways” interconnections. As it offers diverse immune pathways in which specific genes are engaged, IPMAGT encourages researchers to view and interpret the tumor immune microenvironment (TIME) from a more holistic, comprehensive, and integrative perspective.

Second, IMPAGT presents the dysregulation of the immune system even at the tumor budding stage in cervical cancer. “Pathway in Cancer” was the most strongly enriched and activated pathway for both up- and downregulated DEGs. This highlighted the presence of abnormal shifts in gene expression in TB, indicating the activation of carcinogenesis dynamics even from a small cluster of tumor cells. This process supported the initiation of cancer from tumor budding and further revalidated their clinical significance.

In addition, DEG-mapped pathway visualization of “Pathway in Cancer” using KEGG Mapper revealed overactivation of “Signal transduction pathways”, such as the PI3K/Akt, JAK/STAT, and Notch signaling pathways. These signaling pathways eventually pointed toward “Sustained angiogenesis”, “Evading apoptosis”, and “Proliferation”. A detailed investigation of DEG-mapped pathways revealed that up- and downregulated DEGs were mainly involved in the PI3K/Akt signaling pathway. Furthermore, the GO analysis of 133 IMPAGT-overlapped upregulated DEGs revealed that the immune-related genes were directly associated with the characteristics of the PI3K/Akt pathway.

The PI3K/Akt/mTOR signaling pathway is one of the most common aberrantly stimulated pathways in solid tumors [29]. The pathway is critical for processes involved in cancer progression, including cell proliferation, metabolism, apoptosis, and metastasis [30,31,32]. Moreover, this pathway appears to be important for tumor initiation [30,33]. PI3K is a intracellular lipid kinase that is usually activated by receptor tyrosine kinase (RTK) in response to various stimuli, such as growth factors, cytokines, hormones, and insulin [34]. Among three classes of PI3Ks, Class 1A PI3K is the most widely studied in relation to cancer [33]. Class 1A PI3Ks are heterodimers consisting of a catalytic subunit (p110α, p110β, or p110γ) and a regulatory subunit (p85α, p85β, p50α, p55α, or p55γ) [33,34,35,36]. When RTK is activated, regulatory p85 releases p110 catalytic subunits and generates PI3K, which phosphorylates PIP2 to PIP3 [37]. The accumulation of PIP3 on the cell membrane leads to the activation of downstream Akt upon the phosphorylation of Thr308 by PDK1 and Ser473 by mTORC2 [34,38,39]. Subsequently, fully activated Akt phosphorylates multiple downstream target proteins and substrates, such as mTORC1, p21, GSK3, and FOXO, and plays a dominant role in the signal transduction of the entire PI3K signaling pathway [33,37].

The oncogenic stimulation of this pathway is caused by several mechanisms: inappropriate activation of RTK; somatic mutations in PI3KCA, which encodes p110α of PI3K; and the loss of the tumor suppressor PTEN and other molecular signals [33,36,40,41]. PIK3CA is the second most frequently mutated oncogene across cancers, comprising 39% of mutations found in cervical cancer [42,43]. It also has been found that PIK3CA-mutant cervical cancer exhibits poor prognostic features, such as a higher tumor mutation burden and worse survival [31,32,44,45]. PI3K/Akt/mTOR dysregulation in cancer caused by the aforementioned reasons is associated with several cancer hallmarks, such as cell cycle, proliferation, autophagy, apoptosis, angiogenesis and metabolism, metastasis, and the EMT [39,46,47]. As these hallmarks were depicted in our enriched pathway visualization, each point related to the PI3K/Akt/mTOR pathway was examined in more detail.

The PI3K/Akt/mTOR axis is related to antiapoptosis and proliferation in cancer cells. Apoptosis is a programmed cell death pathway that is activated by both the extrinsic death receptor and intrinsic mitochondrial pathways. Peng et al. argued that PI3K/Akt/mTOR signaling leads to the binding of extrinsic death complexes, such as FasL and Fas, which activate caspase 8 and trigger apoptosis with other caspases [46]. Additionally, they mentioned that the balance between proapoptotic (Bad, Bax) and antiapoptotic proteins (Bcl-2, Bcl-x) is lost, thereby promoting the evasion of apoptosis [38,45,46]. Moreover, Akt can negatively regulate transcription factors, such as NF-κB or FOXO, and increase the production of antiapoptotic and prosurvival genes [43,45,48]. In addition to the inhibition of apoptosis, Akt also regulates cell survival through p27, GSK3, or the mTOR complex. To elaborate, Akt regulates the cell cycle via p27 and produces proteins involved in the G1/S phase of the cell cycle, such as cyclin D1, through GSK3 [46,49]. The overactivation of mTORC2 leads to the hyperaccumulation of Akt, which stimulates mTORC1 to phosphorylate translation effectors, such as S6K or 4EBP1, to enhance protein synthesis for cell survival [39,45,46,50].

The PI3K/Akt/mTOR pathway is also critical in sustaining angiogenesis by serving as a master controller of angiogenic signaling in the endothelium [33]. Tumor angiogenesis is caused by an imbalance between proangiogenic factors such as VEGF and antiangiogenic factors such as angiostatin or endostatin [51]. Peng et al. insisted that hypoxia stabilizes HIF-1a and stimulates the PI3K/Akt/mTOR axis in malignant cells [46]. Such activation of the axis boosts VEGF production and enhances vascular permeability, resulting in leaky vessels, sluggish blood flow, and high intestinal pressure, which eventually leads to sustained angiogenesis [46].

Although it was not specifically identified in the DEG-mapped pathway, PI3K/Akt/mTOR signaling is also closely associated with the EMT [52,53]. As mentioned above, epithelial cells change their morphology into a more mesenchymal–fibroblast phenotype. The morphological change is accompanied by decreased intercellular cell–cell adhesion and enhanced motility. Such alteration implies a decreased expression of the epithelial marker E-cadherin and an increased expression of mesenchymal markers, such as vimentin and fibronectin, which are regulated by Snail or Twist signals [10]. Qureshi et al. reported that PI3K signaling is stimulated by EGFR along with hypoxia, and it affects transcription factors, such as Snail and Twist [11]. In addition, NF-κB, mTORC2, and TGF-β are linked to the EMT via the activation of the PI3K/Akt/mTOR signaling pathway [54,55,56,57,58,59,60]. Owing to its importance, several clinical trials have examined the PI3/Akt/mTOR pathway. Several types of inhibitors have been utilized, including pan-PI3K inhibitors targeting all four isoforms of Class 1 PI3K, isoform-selective inhibitors targeting a single isoform of Class 1 PI3K, dual PI3K/mTOR inhibitors, and specific target inhibitors [36,41,42,45]. These small-molecule inhibitors support the inappropriate activation of the PI3K/Akt/mTOR signaling pathway in malignancies in cervical cancer. For example, treatment with rapamycin (mTOR inhibitor) blocks angiogenesis and enhances autophagy in cervical cancer cell lines [46,61]. The PI3K inhibitor LY294002 increases apoptosis and radiosensitization in cervical cancer cell lines [62,63].

Despite the necessity and continuous trials of PI3K inhibitors, only a few treatments have been approved for clinical use for several reasons [64]. First, sufficient doses needed to generate beneficial effects cannot be given to patients because of the risk of “on-target side effects”. Diabetes can be induced by high doses of PI3K inhibitors because the pathway is involved in insulin signaling. In addition, as PI3K/Akt/mTOR is engaged with many effectors, unexpected “off-target side effects” could develop. Furthermore, a recent study proposed a noncanonical PI3K pathway in the nuclei of cells, which is different from the canonical PI3K pathway located in the plasma membrane [65]. Such findings highlight the importance of selective pharmacological inhibition specifically targeting molecules of the PI3K/Akt/mTOR axis without affecting other pharmacodynamics markers or causing unintended potential toxicity [43,66].

On that note, a recently developed technology, termed proteolysis-targeting chimeras (PROTACs), can be used to solve issues related to small-molecule inhibitors. PROTACs are bivalent molecules consisting of a ligand of the protein of interest (POI) and a covalently linked ligand of an E3 ubiquitin ligase. PROTACs recruit E3 ligase in proximity. After binding and forming the complex, the POI is transported to the 26S proteasome for degradation [67,68]. PROTACS repeatedly search for the targeted protein, which facilitates POI elimination at extremely low doses [69,70]. As PROTACs specifically eliminate POIs with better selectivity, low toxicity, and fewer on-/off-target side effects, they may be adapted as clinically useful treatments [71]. Additional studies of PROTACs targeting PI3K/Akt/mTOR signaling pathways are being conducted [70,72].

The limitations of our study include the small number of samples with pathway-level results. Because the current data focused on the most enriched pathway related to the TIME of TB in cervical cancer, specific details, such as gene-level biomarkers or specific aspects of molecular reaction in the PI3K/Akt/mTOR pathway, have not been elucidated. However, these issues can be resolved by planned follow-up research on PI3K PROTACs with more clinical samples and validation studies in cervical cancer cell lines. Moreover, protein-level analyses, such as Immunohistochemistry and Western blotting, are planned in the next follow-up study as well. Despite these limitations, our study validated the presence of immune dysregulation in TB of cervical cancer, especially the dysregulation of the PI3K/Akt/mTOR signaling pathway. In addition, the findings emphasized the clinical significance of tumor budding in relation to tumorigenesis. Furthermore, the study asserted the necessity of in-depth investigations on the PI3K/Akt/mTOR axis to develop more specifically targeted patient-tailored treatments in cervical cancer.

## 5. Conclusions

The newly generated database IMPAGT revealed the existence of immune dysregulation dynamics and the specific enrichment of the PI3K/Akt/mTOR signaling pathway in the tumor immune microenvironment of cervical cancer with high tumor budding. Further research focused on the PI3K/Akt/mTOR axis and selective therapeutic approaches targeting this pathway could help develop better targeted therapy strategies for cervical cancer.

## Figures and Tables

**Table 1:** Clinicopathologic characteristics of 21 cervical cancer samples. All 21 patients were staged following revised 2009 FIGO (International Federation of Gynecology and Obstetrics) staging system. (n, %) indicates the numbers and percentage of samples in each parameter out of total 21.

Variables	N = 21
Age (years)	47.4 ± 11.0
FIGO stage (n, %)	
IB1	11 (52.4)
IB2	2 (9.5)
IIA2	4 (19.0)
IIB	4 (19.0)
Histology (n, %)	
Squamous cell carcinoma	15 (71.4)
Adenocarcinoma	5 (23.8)
Adenosquamous carcinoma	1 (4.8)
HPV type (n, %)	
HPV 16	16 (52.4)
HPV 18	1 (4.8)
HPV 31	1 (4.8)
HPV 33	1 (4.8)
HPV 35	1 (4.8)
Negative infection	3 (14.3)
Unknown	5 (23.8)
Primary tumor size (cm)	3.3 ± 1.6
Lymphovascular invasion (n, %)	15 (71.4)
Deep stromal invasion (n, %)	18 (85.7)
Parametrial invasion (n, %)	8 (38.1)
Positive vaginal margin (n, %)	1 (4.8)
Lymph node metastasis (n, %)	11 (52.4)
High tumor budding (≥3, n, %)	15 (71.4)
Recurrence (n, %)	6 (28.6)
Death (n, %)	4 (19.0)

FIGO = International Federation of Gynecology and Obstetrics; HPV = human papilloma virus.

**Table 2:** A table of significantly enriched pathways. Pathways with more than 10 DEGs were considered as significantly enriched pathways. A list of significantly enriched pathways from up- and downregulated DEGs are presented with KEGG pathway ID and numbers of DEGs involved in each pathway.

Up/Down DEGs	KEGG Pathway ID	Pathway	Number of DEGs Involved
Up	Hsa 05200	Pathway in Cancer	20
	Hsa 04612	Antigen processing and presentation	13
	Hsa 05202	Transcriptional misregulation in Cancer	13
	Hsa 04010	MAPK Signaling Pathway	12
	Hsa 04621	NOD-like receptor signaling Pathway	12
	Hsa 04514	Cell adhesion molecules	11
	Hsa 04060	Cytokine-cytokine receptor interaction	10
	Hsa 04151	PI3K/Akt signaling pathway	9
Down	Hsa 05200	Pathway in Cancer	10

**Table 3:** Significant BP terms of 133 upregulated DEGs overlapped with IMPAGT.

GO ID	BP Terms	*p*-Value
GO: 0007165	Signal transduction	1.3 × 10^−4^
GO: 0006955	Immune response	9.8 × 10^−10^
GO: 0008284	Positive regulation of cell proliferation	1.3 × 10^−2^
GO: 0043065	Positive regulation of apoptotic process	7.2 × 10^−^^3^
GO: 0042981	Regulation of apoptotic process	6.0 × 10^−3^
GO: 0042127	Regulation of cell proliferation	5.2 × 10^−^^3^
GO: 0001934	Positive regulation of protein phosphorylation	1.4 × 10^−2^

## Data Availability

Not applicable.

## Supplementary Materials

The following supporting information can be downloaded at: https://www.mdpi.com/article/10.3390/cimb44110350/s1, Table S1: List of 547 gene symbols of LM22; Table S2: IMPAGT and IMPAGT Gene Set.
